# [^68^Ga]Ga-PSMA-11: The First FDA-Approved ^68^Ga-Radiopharmaceutical for PET Imaging of Prostate Cancer

**DOI:** 10.3390/ph14080713

**Published:** 2021-07-23

**Authors:** Ute Hennrich, Matthias Eder

**Affiliations:** 1 German Cancer Research Center (DKFZ), Division of Radiology, 69120 Heidelberg, Germany; 2 Division of Radiopharmaceutical Development, German Cancer Consortium (DKTK), Partner Site Freiburg, and German Cancer Research Center (DKFZ), 69120 Heidelberg, Germany; matthias.eder@uniklinik-freiburg.de; 3 Department of Nuclear Medicine, University Medical Center Freiburg, Faculty of Medicine, University of Freiburg, 79106 Freiburg, Germany

**Keywords:** [^68^Ga]Ga-PSMA-11, PSMA, prostate cancer, theranostics, positron emission tomography (PET)

## Abstract

For the positron emission tomography (PET) imaging of prostate cancer, radiotracers targeting the prostate-specific membrane antigen (PSMA) are nowadays used in clinical practice. Almost 10 years after its discovery, [^68^Ga]Ga-PSMA-11 has been approved in the United States by the Food and Drug Administration (FDA) as the first ^68^Ga-radiopharmaceutical for the PET imaging of PSMA-positive prostate cancer in 2020. This radiopharmaceutical combines the peptidomimetic Glu-NH-CO-NH-Lys(Ahx)-HBED-CC with the radionuclide ^68^Ga, enabling specific imaging of tumor cells expressing PSMA. Such a targeting approach may also be used for therapy planning as well as potentially for the evaluation of treatment response.

## 1. Introduction

Nowadays, radiotracers that target the prostate-specific membrane antigen (PSMA) are used in clinical practice for the positron emission tomography (PET) imaging of prostate cancer. On 1 December 2020, almost 10 years after its discovery, [^68^Ga]Ga-PSMA-11 was approved by the Food and Drug Administration (FDA) [1] as the first ^68^Ga-labeled radiopharmaceutical for PET imaging of PSMA-positive prostate cancer. Holders of marketing authorizations are the University of California Los Angeles (UCLA) Biomedical Cyclotron Facility (Los Angeles, CA, USA) and the University of California San Francisco (UCSF) Radiopharmaceutical Facility (San Francisco, CA, USA).

PSMA is expressed as a type II transmembrane protein on the surface of prostate cancer cells. The protein shows a moderate to low expression on healthy tissue, whereas normal prostate cells show the highest background expression levels in the body. According to the biological role of PSMA in certain cellular processes, PSMA is also known as glutamate carboxypeptidase II (GCPII) or folate hydrolase 1 (FOLH1), indicating the metalloenzymatic function that hydrolyzes carboxyterminated glutamates on, for example, polyglutamated folate molecules and other structures. Due to the fact that normal prostate cells and prostate cancer cells express the highest levels of PSMA in the body, the overall expression profile is highly favorable and renders low molecular weight inhibitors of PSMA suitable for the diagnosis and therapy of prostate cancer. Several studies have shown that PSMA expression on prostate cancer is prognostic in terms of Gleason score, probability of recurrence, or progression [2,3,4]. The aggressiveness of prostate cancer is associated with a correlation of PSMA expression and other factors [5]. Consequently, there has been a significant level of research activity in this field, and the first PSMA inhibitors for Nuclear Medicine applications have been explored extensively for many years [6,7]. Besides the anti-PSMA antibody J591 [8], the development of PET imaging agents has mainly focused on urea-based small-molecule inhibitors of PSMA. Prominent examples in the field of PSMA PET/CT imaging of prostate cancer include [^18^F]DCFBC [9], [^18^F]DCFPyL [10], [^18^F]PSMA-1007 [11,12], and the herein discussed compound, [^68^Ga]Ga-PSMA-11 [13]. 

**Figure 1:**
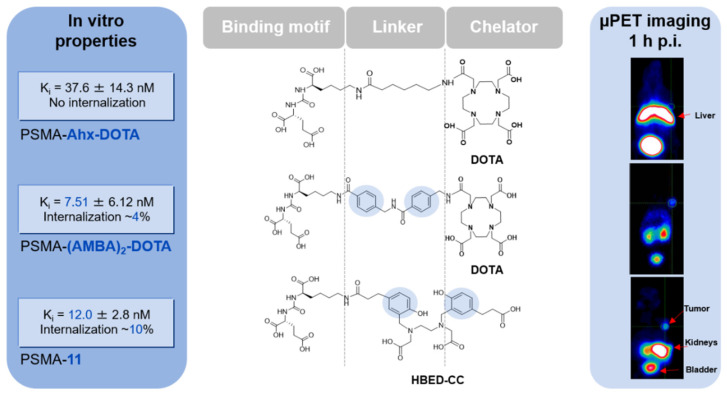
The pharmacokinetic and binding properties of ^68^Ga-labeled PSMA targeting urea-based inhibitors are clearly affected by the exact composition of the linker and the presence of aromatic moieties. In case the linker is entirely aliphatic (PSMA-Ahx-DOTA), the affinity and internalization are low or lost, respectively, and the tracer is mainly localized in the liver without any detectable signal in the tumor. By introducing aromatic moieties in the linker region as represented by PSMA-(AMBA)_2_-DOTA, the compound exhibits better affinity and starts to show significant internalization of the tracer in the tumor cell, resulting in a visible tumor uptake and a changed PK profile. One of the unique characteristics of PSMA-11 is that the chelator HBED-CC takes over these functional requirements by its aromatic moieties and, therefore, interacts well with the binding pocket, resulting in a promising in vivo performance of the entire molecule. Data were obtained from previously published studies using LNCaP cells for the determination of affinity and internalization and LNCaP-tumor-bearing BALB/c nu/nu mice for PET imaging [13,14].

The discovery process of PSMA-11 clearly emphasizes the complex characteristics of the PSMA binding site, requiring defined lipophilic interactions within the PSMA active-site flanking funnel (Figure 1). It has been shown that aromatic moieties in the linker region of urea-based PSMA inhibitors can dramatically change the internalization properties and, accordingly, the tumor uptake [14]. As *N*,*N*′-bis[2-hydroxy-5(carboxyethyl)benzyl]ethylenediamine-*N*,*N*′-diacetic acid (HBED-CC) exhibits two aromatic moieties, it is introduced as a linker and chelator in the urea-based inhibitor. Hence, HBED-CC is simultaneously acting as a radiometal chelator for the incorporation of ^68^Ga and as a functional moiety triggering internalization and tumor uptake [13]. In other words, PSMA-11 represents a unique example of radiometal-labeled radiopharmaceuticals consisting solely of the pharmacophore while still being able to complex ^68^Ga.

In 2011, this low-molecular-weight variant was first used as a PET imaging agent in patients [15] and thereafter has been successfully implemented as a leading radiopharmaceutical for the PET/CT imaging of recurrent prostate cancer. The radiotracer has been comprehensively studied, and the results show that PSMA PET/CT outperforms any other currently available imaging modality for prostate cancer. In particular, in combination with targeted radionuclide therapy, PSMA imaging has been proposed for patient selection and monitoring and has been established as an ideal representative of the theranostic concept [16]. [^68^Ga]Ga-PSMA-11 PET/CT is able to accurately localize and help define the extent of primary prostate cancer [17]. In radiation oncology, [^68^Ga]Ga-PSMA-11 has been extensively explored since it represents another field that relies on risk stratification and exact staging. In 2016, [^68^Ga]Ga-PSMA-11-based PET imaging was proposed as a key technology for individualized radiotherapy in prostate cancer patients as it was reported that the therapeutic strategy was changed in 50.8% of the cases [18]. There might even be an impact of [^68^Ga]Ga-PSMA-11 PET/CT on radiotherapeutic concepts for patients with primary prostate cancer [19,20,21], whereas a major focus of PSMA-11-related research has been reported in the field of recurrent prostate cancer. In numerous partly multicentric retrospective studies, [^68^Ga]Ga-PSMA-11 PET imaging has significantly improved the detection of recurrent prostate cancer as compared with conventional imaging or formerly used choline-based PET imaging [22,23,24,25,26]. A prospective single-arm clinical trial confirmed these observations as disease localization by [^68^Ga]Ga-PSMA-11 PET imaging led to therapy management changes in more than half of patients with biochemical recurrence [27]. A significant impact on the management of prostate cancer patients in other clinical scenarios besides biochemical recurrence and presurgical staging has been demonstrated in another prospective clinical trial [28].

Taken together, [^68^Ga]Ga-PSMA-11 PET imaging has a huge potential in many clinical scenarios of prostate cancer diagnosis and therapy. This is further emphasized by several clinical guidelines published in the Journal of Nuclear Medicine or the European Journal of Nuclear Medicine and Molecular Imaging [29,30]. The harmonization of protocols for [^68^Ga]Ga-PSMA-11 imaging led by the Clinical Trials Network of the Society of Nuclear Medicine and Molecular Imaging served as a basis for the final FDA approval of the drug. In the FDA approval, [^68^Ga]Ga-PSMA-11 is indicated for the initial diagnosis and staging of prostate cancer patients with suspected metastases and the imaging of patients with suspected biochemical recurrence after prostatectomy or radiation therapy. From a radiopharmaceutical point of view, [^68^Ga]Ga-PSMA-11 has special characteristics and requirements attributed to the radiochemical properties of ^68^Ga. Due to the relatively short physical half-life of ^68^Ga (T_1/2_ = 67.7 min), a satellite distribution of this tracer is not feasible: it has to be prepared on-site or at least nearby. With regard to the FDA approval, only facilities in the near vicinity of Los Angeles and San Francisco may profit from buying the ready-to-use injection solution. Other facilities have to produce their own tracer batches on-site with all legal consequences involved in a radiotracer production for human application. However, due to the existing FDA approval, other hospitals in the US might apply for an abbreviated NDA (new drug application) approval in an accelerated process [31].

## 2. Chemical Overview

### 2.1. Names and Chemical Structure of [^68^Ga]Ga-PSMA-11

Another name for [^68^Ga]Ga-PSMA-11 is [^68^Ga]Ga-PSMA-HBED-CC, and its sequence is [^68^Ga]Ga-HBED-CC-Ahx-Lys(OH)-CO-Glu(OH). The complete IUPAC name of [^68^Ga]Ga-PSMA-11 is as follows:

[^68^Ga]gallium (3S,7S)-22-[3-[[[2-[[[5-(2-carboxyethyl)-2-hydroxyphenyl]-methyl](carboxymethyl)amino]ethyl](carboxymethyl)amino]-methyl]-4-hydroxyphenyl]-5,13,20-trioxo-4,6,12,19-tetraazadocosane-1,3,7-tricarboxylic acid.

**Figure 2:**
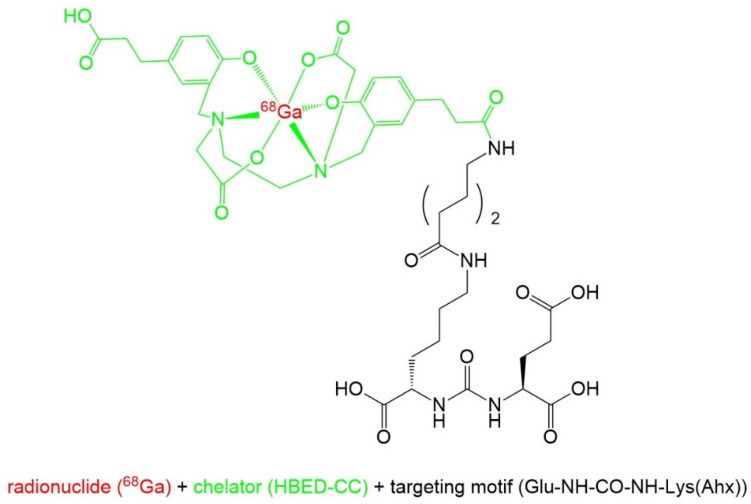
Chemical structure of [^68^Ga]Ga-PSMA-11.

Complexation of the radionuclide gallium-68 is achieved by the bifunctional acyclic chelator HBED-CC (*N*,*N*′-bis[2-hydroxy-5-(carboxyethyl)benzyl]ethylenediamine-*N*,*N*′-diacetic acid), which is a hexadentate chelator with octahedral geometry. In this complex, gallium-68 is coordinated to two nitrogen atoms, two hydroxy groups, and two carboxylic groups [32,33]. The [^68^Ga]Ga-HBED-CC group is bound to the PSMA affine peptidomimetic Glu-NH-CO-NH-Lys(Ahx) [13]. Figure 2 shows the structure of [^68^Ga]Ga-PSMA-11.

### 2.2. Gallium-68

With a half-life of 67.7 min, the radionuclide gallium-68 (^68^Ga) decays through positron emission to the stable isotope zinc-68 (^68^Zn) [34]. Further decay characteristics of gallium-68 are a positron yield of 89.1%, a maximal β^+^ energy of 1899 keV, and an average β^+^ energy of 836 keV. An important parameter for the quality of a PET image is the mean positron range in soft tissue, which is 1.05 mm [35].

For the production of commonly used PET nuclides, such as fluorine-18 and carbon-11, a cyclotron is needed, which may not be available at every site. Especially for these sites, the use of ^68^Ga-labeled radiotracers is very attractive because it is available by elution of ^68^Ge/^68^Ga generators. These generators can be purchased from different companies and in different capacities and grades, for research and GMP (good manufacturing practice) purposes. In case of patient application, the production of the ^68^Ga-labeled radiopharmaceutical has to be conducted under GMP conditions necessitating the use of an authorized generator. In Europe, the authorized generators are GalliaPharm^®^ (Eckert & Ziegler, Berlin, Germany) and Galli Ad^®^ (IRE ELit, Fleurus, Belgium), while in the US, the FDA-approved versions GalliaPharm^®^ (Eckert & Ziegler, Berlin, Germany) and Galli Eo^®^ (distributed by Cardinal Health, Dublin, OH, USA) are available. The parent nuclide germanium-68 (^68^Ge; half-life 271 d) continuously decays via electron capture (EC) to its daughter nuclide ^68^Ga, which can be eluted multiple times a day using diluted hydrochloric acid (regeneration needs approximately 3 half-lives of ^68^Ga) [36]. If a reduction of the procurable radioactivity to less than 1/2 and the associated decline of patient doses is acceptable, one generator can be used for up to 1 year. Safe usage of the generator without ^68^Ge breakthrough for this period of time is guaranteed by the vendors. Due to the typical capacity of ^68^Ge/^68^Ga generators (1.85 GBq), the producible amount of radioactivity might be rather low (e.g., in comparison with ^18^F tracers), therefore limiting the number of patients that can be imaged with one batch. This disadvantage, however, might be overcome by repeated tracer productions per day. A problem can be the very high demand for authorized ^68^Ge/^68^Ga generators, which may result in long delivery times and increased prices (up to approx. USD 100,000 per generator). To circumvent these disadvantages, a large-scale production of ^68^Ga using a cyclotron is currently being investigated [37], which might be a useful alternative for facilities with an on-site cyclotron. Cyclotron-produced ^68^Ga may be obtained by irradiation of either a liquid or a solid target containing enriched Zinc-68. Appropriate targets and automated purification solutions are commercially available from common cyclotron vendors (e.g., GE Healthcare, Waukesha, WI, USA, and IBA Radiopharma Solutions, Louvain-la-Neuve, Belgium). Using a liquid target of up to 9.85 GBq of ^68^Ga might be achievable, while using a solid target, it might be up to 194 GBq [38]. Recent studies have also evaluated the production of ^68^Ga-labeled tracers (e.g., [^68^Ga]Ga-PSMA-11) with cyclotron-produced ^68^Ga and obtained approximately 1.65 GBq of end product (liquid target production [39]) and up to 72.2 GBq of end product (solid target production [40]), respectively. The recognition of the cyclotron production of ^68^Ga as an accepted manufacturing route is further demonstrated by the recent release of the European Pharmacopoeia monograph “Gallium (^68^Ga) chloride (accelerator-produced) solution for radiolabeling” (01/2021:3109) [41] and by the FDA approval of the production of [^68^Ga]Ga-PSMA-11 using cyclotron-produced ^68^Ga at UCSF. Considering all facts, ^68^Ga has a great potential and can compete with the most used PET isotope, fluorine-18.

### 2.3. Production and Quality Control of [^68^Ga]Ga-PSMA-11

Ready-to-use [^68^Ga]Ga-PSMA-11 injection solution is commercially available by the UCLA Biomedical Cyclotron Facility (Los Angeles, CA, USA) and the UCSF Radiopharmaceutical Facility (San Francisco, CA, USA), but due to the limited half-life of ^68^Ga, this can only be distributed in the near vicinity, and only a few sites may profit from this. For the on-site preparation of [^68^Ga]Ga-PSMA-11 in clinics, generally, two different methods are possible: a GMP-compliant synthesis using an automated synthesis module in a clean room, which has to be approved by local authorities, and the use of so-called “cold kits”, allowing tracer production without a dedicated clean room.

The most common way of PET tracer production is the use of an automated synthesis. For this purpose, a lot of different synthesis modules from various vendors are on the market, most of them nowadays cassette based. This approach facilitates the production of different tracers on one module because the cleaning of the module after production along with the validation of the cleaning procedure is no longer necessary: after synthesis, the cassette with all its reagent pathways is just disposed of, and for a new production, a new one is installed. For the production of [^68^Ga]Ga-PSMA-11, GMP-compliant kits for various different modules are commercially available. At our site, synthesis of [^68^Ga]Ga-PSMA-11 was routinely carried out on a GRP module (att Scintomics GmbH, Fürstenfeldbruck, Germany) along with the GalliaPharm^®^ generator, producing far more than 100 batches over the years. In this case, the eluate of the ^68^Ge/^68^Ga generator is first trapped on a cation exchange cartridge and then eluted with sodium chloride solution into a reaction vial containing the labeling precursor PSMA-11 (10 µg) dissolved in acetate buffer and ascorbate. Reaction takes place at 100 °C for 10 min, and then the product is purified on a C18 cartridge. The purified product is eluted with an ethanol/water mixture, diluted with phosphate-buffered saline, and sterile-filtered. The overall production time is 30 min. Fulfilling GMP compliance, all reaction steps have to take place in a class C clean room environment, and sterile filtering and portioning of the product in a class A environment, but this may differ from country to country. In order to support other US sites applying for an abbreviated NDA approval from the FDA, the UCLA and UCSF teams have published key details of their application [31]. The production methods for [^68^Ga]Ga-PSMA-11 differ at both sites (e.g., while at UCLA only a generator is used for obtaining ^68^Ga, at UCSF also the production of ^68^Ga with a cyclotron is approved). It is not clear from the description of the production whether full GMP compliance is necessary.

A much easier and faster way to produce [^68^Ga]Ga-PSMA-11 may be the use of a sterile cold kit, a technology that has been in use for the preparation of ^99m^Tc tracers for a long time. For the production of [^68^Ga]Ga-PSMA-11, two different cold kits are commercially available, one from Telix Pharmaceuticals (TLX591-CDx, Illumet^TM^ or Illuccix^®^; Melbourne, Australia) [42] and one from Isotopia Molecular Imaging Ltd. (isoPROtrace-11; Petach Tikva, Israel) [43]. Using these cold kit preparations, the radiotracer can be easily prepared without the need for a clean room. Both kits can be used with the commercially available and authorized ^68^Ge/^68^Ga generators. Using the cold kit TLX591-CDx, ^68^Ga has to be eluted first into a separate sterile vial before the addition of the dissolved labeling precursor and reaction for 5 min at room temperature [44]. While the kit by Telix Pharmaceuticals consists of three vials, the kit by Isotopia consists of only one vial, further facilitating the convenient synthesis [45]. Using the isoPROtrace-11 kit, [^68^Ga]Ga-PSMA-11 can be prepared by just adding the ^68^Ga eluate to a sterile kit vial containing the lyophilized labeling precursor and reacting for 5 min at room temperature. The quality of the product has been validated for both cold kit preparations, and its clinical applicability has been demonstrated [44,46]. One drawback of the kit preparations is that they have not yet been approved by international authorities and can only be used in investigational studies. However, both companies have applied for approval by international authorities.

In order to ensure patient safety, the quality of each radiopharmaceutical batch has to be checked before application. Depending on the local authorities, the amount of required analyses may differ. In Europe, the acceptance criteria and suitable analytical methods for [^68^Ga]Ga-PSMA-11 are described in the European Pharmacopoeia monograph “Gallium-68 PSMA-11 injection solution” (04/2021: 3044) [47]. These acceptance criteria have to be evaluated for each batch; otherwise, it has to be explained carefully why some of the parameters are (partly) not analyzed. Table 1 summarizes the acceptance criteria proposed in the monograph.

All analytical methods have to be validated in compliance with GMP regulations before use. For the determination of the chemical and radiochemical purity of [^68^Ga]Ga-PSMA-11, a suitable HPLC method including UV and radioactivity detection may be used. Depending on the temperature, time, and pH of the solution, diastereomers of [^68^Ga]Ga-PSMA-11 might be formed, detectable as three peaks in the radiochromatogram. In a preclinical study, it was demonstrated in cell experiments that the diastereomers do not bind differently to the PSMA receptor, and therefore, the PET imaging should not be influenced [33]. Usually, two peaks corresponding to the thermodynamically most stable diastereomer and another diastereomer are seen, and their combined peak areas should be more than 95% of the detected radioactivity. Another important parameter of the injection solution is its chemical purity with respect to PSMA-11 (labeling precursor) and other related substances that might be present. The determination of their content might be performed by the evaluation of their peak areas in comparison with the peak area of the reference standard PSMA-11. Since the product solution may also contain unreacted [^68^Ga]Ga^3+^, it is of utmost importance to evaluate its content. In case of its colloidal form, this can only be done by thin-layer chromatography (TLC) because this form does not elute from the HPLC column. Depending on the production method, other important parameters related to the chemical purity of the injection solution may be its ethanol content and the content of HEPES if it is used as a buffer in the labeling reaction. Additionally, the pH value and the content of bacterial endotoxins and the sterility (can only be determined postrelease) of the injection solution should be part of the batch quality control. On the basis of a risk assessment, it has to be defined together with the local authorities which tests have to be conducted for validation only, periodically, or for every batch. For example, the FDA approval does not require the determination of chemical and radiochemical purity by HPLC for each batch [31]. When using the sterile cold kit methodology for production, it is to be expected that only a very limited quality control (e.g., TLC and pH value) will be required.

## 3. Medicinal and Pharmaceutical Overview

### 3.1. Clinical Indication

[^68^Ga]Ga-PSMA-11 is a diagnostic radiotracer for the localization of PSMA-positive lesions in men with prostate cancer using PET [48]. It is indicated in cases of suspected metastases for initial definitive therapy and in cases of suspected recurrence based on elevated serum prostate-specific antigen (PSA) levels.

### 3.2. Application

In the prescribing information of [^68^Ga]Ga-PSMA-11, the subsequent application recommendations are given [48]. The recommended amount of radioactivity for an intravenous bolus injection of [^68^Ga]Ga-PSMA-11 is between 111 and 259 MBq in adults. After injection of the radiotracer, an intravenous flush of sterile physiological saline solution (0.9%) should be administered. If not contraindicated, a diuretic might be given at the time of radiotracer application in order to decrease artifacts from its accumulation in the urinary bladder and ureters. Patients should drink water to ensure adequate hydration, also following radiotracer administration, to reduce radiation exposure by frequent voiding. Imaging can be started at 50 min postinjection (p.i.) (range 50–100 min), beginning the scan caudally and proceeding cranially. Immediately prior to scanning, the patient should void.

### 3.3. Pharmacology and Pharmacokinetics

All urea-based PSMA inhibitors typically show a very similar and characteristic biodistribution. The first reported biodistribution related to PSMA-11 was published in 2012. In 37 patients, a typical and high physiologic PSMA ligand uptake was found in the kidneys and salivary glands, whereas the lacrimal glands, liver, spleen, and bowel revealed a low to moderate uptake [49]. As PSMA is acting as a metalloenzyme that hydrolyzes polyglutamated folate molecules and other molecules with carboxyterminated glutamates, the physiological uptake is often attributed to a certain biological function and expression in several human tissues, particularly within the brain and the small intestine. The normal prostate gland presents with a significant but low uptake (SUV_mean_ of 2.9) of [^68^Ga]Ga-PSMA-11, which is clearly delineable from local relapses of prostate cancer [49]. Background uptakes are mainly caused by a physiological expression of PSMA in the respective tissues, whereas the high uptake of PSMA inhibitors in the salivary glands is not entirely based on PSMA-specific mechanisms. In several preclinical investigations, also a nonspecific uptake of PSMA inhibitors has been observed in this tissue [50,51]. In particular, with regard to radionuclide therapy, the salivary gland uptake might cause side effects, such as xerostomia, and has been reported as the dose-limiting organ during alpha therapy of prostate cancer [52].

In a study by Afshar-Oromieh et al. on the dosimetry of PSMA-11 PET, it was found that the mean effective dose of a [^68^Ga]Ga-PSMA-11 PET scan is 0.023 mSv/MBq, resulting in a typical average effective dose of 4.7 mSv per scan when 200 MBq is injected [53]. The kidneys receive the highest absorbed dose with an average dose of 0.262 mGy/MBq (Table 2), however, without any dose-limiting impact in PSMA-11-related diagnosis.

The optimal imaging time point has been reported to be at 1 h postinjection as nearly all lesions appear with sufficient contrast. However, radioactivity accumulates further, typically resulting in a better imaging contrast at 3 h postinjection. In specific clinical scenarios, it might therefore be advantageous to examine the patient at later time points [53].

Due to the excellent specificity and low background of PSMA ligands, a pathologic PSMA uptake typically presents with a high tumor-to-background contrast and allows the identification of a small-volume nodal or visceral lesion. Any focal uptake not associated with a physiologic uptake is therefore considered potentially pathologic. The typical metastatic site is the pelvic region, often followed by distant lymph nodes and bone metastases. A prospective analysis of the accuracy of [^68^Ga]Ga-PSMA-11 PET/CT by Hofman et al. revealed an overall sensitivity of 85% (vs. 38%) and a specificity of 98% (vs. 91%), outperforming conventional imaging in prostate cancer (bone scan/CT) [54].

## 4. Perspective

PSMA inhibitors have gained increasing interest as radiopharmaceuticals for molecular imaging. To date, several small-molecule compounds targeting PSMA have been developed for PET imaging in prostate cancer, showing an impact in many medical fields, such as radiology, radiation oncology, targeted radionuclide therapy, and intraoperative navigation. High sensitivity has been reported to have significant clinical implications for treatment changes at various stages of prostate cancer. For instance, a significant impact on radiotherapy planning has been demonstrated in several studies, resulting in substantial changes in patient management in more than half of the patients with biochemical recurrence of prostate cancer [27]. The observed management changes have mainly been focal or salvage therapy in the case of local disease and systemic treatment in the case of newly diagnosed distant metastases [17]. Future randomized trials are in preparation to evaluate the impact of management changes on further oncologic outcome parameters.

For targeted radionuclide therapy, the methodology can help to determine the receptor expression and therapy success. Additionally, the field of fluorescence-guided surgery will provide novel clinical concepts in the upcoming years. In urology or surgery, PSMA PET and intraoperative fluorescence using the same molecule might help to identify malignant tissue. Recently, a novel hybrid tracer based on PSMA-11 has been reported for fluorescence-guided surgery in prostate cancer [55], and future work is expected to considerably improve lymphadenectomy. PSMA-11 imaging might also have a significant role in primary diagnosis, as demonstrated in a more recent study by Fendler et al. [17]. [^68^Ga]Ga-PSMA-11 PET/CT identified positive regions within the prostate with a sensitivity of 67% and a specificity of 92% in patients with biopsy-proven prostate cancer, thereby suggesting a potential future role of PSMA-11 in PET-guided biopsy. Thus, intraprostatic tumor localization has been shown to be feasible using [^68^Ga]Ga-PSMA-11. In another study, the imaging findings were further correlated with histopathology and demonstrated significantly higher [^68^Ga]Ga-PSMA-11 uptake in positive segments as compared with negative segments, emphasizing again the high sensitivity and specificity in the detection of primary prostate cancer [21]. In particular, in combination with multiparametric MRI, [^68^Ga]Ga-PSMA-11 imaging clearly demonstrates a great potential for biopsy guidance [56].

Perspectively, ^18^F-labeled PSMA inhibitors will also be of high value, in particular considering radiopharmaceutical aspects. Due to its longer half-life, ^18^F is the radionuclide of choice and enables central manufacturing and distribution of the radiopharmaceutical. Studies evaluating ^18^F-labeled PSMA ligands suggest a similar performance as compared with [^68^Ga]Ga-PSMA-11. In metastatic prostate cancer patients, the diagnostic performances of both [^18^F]PSMA-1007 PET/CT and [^18^F]DCFPyL PET/CT are also highly promising for accurate local staging in recurrent disease and has been shown to be at least equivalent to [^68^Ga]Ga-PSMA-11 [57,58,59,60].

## 5. Conclusions

According to the FDA approval, [^68^Ga]Ga-PSMA-11 is indicated for patients with suspected prostate cancer metastasis who are potentially curable by surgery or radiation therapy, as well as for patients with suspected prostate cancer recurrence. Several studies could show a major impact of PSMA PET imaging on the clinical management of prostate cancer, thereby demonstrating increased accuracy, specificity, and sensitivity compared with conventional imaging. The significantly improved detection rate of lesions in patients with low PSA values supports individualized treatment and therapy in various medical fields.

## Figures and Tables

**Table 1:** Acceptance criteria for [^68^Ga]Ga-PSMA-11 injection solution based on the European Pharmacopoeia monograph [47].

Parameter	Acceptance Criteria
Appearance	Clear, colorless solution
Radiochemical Identity	Similar retention time to Ga-PSMA-11 reference standard
Radiochemical Purity	HPLC ≥ 95%
	TLC [^68^Ga]Ga^3+^ ≤ 3%
Chemical Purity—PSMA-11 Related	PSMA-11 < 30 µg/V_max_^1^
	Sum of related impurities ^2^ ≤ area of reference peak ^3^
	Disregard limit for peak areas ≤ 0.1 × area of reference peak ³
Chemical Purity—Other Substances	HEPES ^4^ ≤ 500 µg/V_max_^1^
	Ethanol ≤ 10% V/V
pH Value	4–8
Endotoxin Content	175/V_max_^1^ IU/mL
Radionuclidic Purity	Half-life, 61–74 min
	Gammaspectroscopy ^5^ 511, 1022, and 1077 keV lines
	90–110% of declared ^68^Ga radioactivity
Sterility ^5^	Sterile

^1^ V_max_: maximum recommended injection volume per patient; ^2^ sum of area of peaks with relative retention of 0.8 to 1.3 in reference to PSMA-11; ^3^ peak area of reference solution PSMA-11 (30 µg/V_max_) has to be determined for comparison; ^4^ HEPES: 2-[4-(2-hydroxyethyl)piperazin-1-yl]ethanesulfonic acid, which may be used as buffer in the labeling reaction; ^5^ evaluation postrelease.

**Table 2:** Absorbed organ doses of [^68^Ga]Ga-PSMA-11 in selected organs [53].

Organ	Absorbed Dose (mGy/MBq) Mean (±SD)
Small Intestine	0.0163 (±0.0022)
Upper Colon	0.0540 (±0.0416)
Kidneys	0.2620 (±0.0984)
Liver	0.0309 (±0.0042)
Muscle	0.0105 (±0.0004)
Red Marrow	0.0092 (±0.0003)
Spleen	0.0446 (±0.0209)
Testes	0.0104 (±0.0006)
Urinary Bladder	0.1300 (±0.0341)
Total Body	0.0124 (±0.0004)
Effective Dose (mSv/MBq)	0.0230 (±0.0036)

## Data Availability

Data sharing not applicable.
